# Identification of non-coding RNA related prognosis biomarkers based on ceRNA network in thyroid cancer

**DOI:** 10.3389/fgene.2023.1157438

**Published:** 2023-04-20

**Authors:** Xin Fang, Xiliang Chen, Jingquan Gao, Liquan Tong

**Affiliations:** ^1^Department of General Surgery II, Daqing Oilfield General Hospital, Daqing, China; ^2^Department of Rehabilitation, Beijing Rehabilitation Hospital of Capital Medical University, Beijing, China; ^3^Department of Nursing Sciences, Faculty of Medicine and Health, Lishui University, Lishui, China; ^4^Department of General Surgery, The Fifth Affiliated Hospital of Harbin Medical University, Daqing, China

**Keywords:** thyroid cancer, prognosis marker, miRNAs, lncRNA, network

## Abstract

**Introduction:** Thyroid cancer (THCA) has become a serious malignant tumor worldwide. Identification of non-coding RNA related regulators is very necessary to improve the knowledge of THCA treatment. The aim of this study was to identify novel therapeutic targets and prognosis biomarkers for predicting pathological characteristics and subsequently treating THCA.

**Methods:** We investigated the alterations of miRNAs, mRNAs and lncRNAs in THCA. Functional enrichment and clustering analysis were conducted for these aberrantly expressed RNAs. Multiple interaction networks among miRNAs, mRNAs and lncRNAs were constructed and the functional modules associated with THCA patients’ prognosis were identified. Furthermore, we evaluated the prognostic roles of the important miRNAs, mRNAs and lncRNAs in THCA and investigated the regulatory potential of non-coding RNAs on immune cell infiltration.

**Results:** We firstly identified that *miR-4709-3p* and *miR-146b-3p* could significantly classify patients into high/low risk groups, which may be potential prognosis biomarkers of THCA. Secondly, we constructed a THCA-related miRNA-mRNA network, which displayed small world network topological characters. Two THCA-related functional modules were identified from the miRNA-mRNA network by MCODE. Results showed that two modules could implicate in known cancer pathways, such as apoptosis and focal adhesion. Thirdly, a THCA-related miRNA-lncRNA network was constructed. A subnetwork of miRNA-lncRNA network showed strong prognosis effect in THCA. Fourthly, we constructed a THCA-related mRNA-lncRNA network and detected several typical lncRNA-miRNA-mRNA crosstalk, such as *AC068138*, *BCL2*, *miR-21* and *miR-146b*, which had good prognosis effect in THCA. Immune infiltration results showed that lncRNAs LA16c−329F2, RP11−395N3, RP11−423H2, RP11−399B17 and RP11–1036E20 were high related to neutrophil and dendritic cell infiltration.

**Discussion:** Non-coding RNA-mediated gene regulatory network has the strong regulatory potential in pathological processes of THCA. All these results could help us uncover the non-coding RNA-mediated regulatory mechanism in THCA.

## Introduction

Thyroid cancer (THCA) is the most common malignant disease of the endocrine system, which usually originates from thyroid epithelial cells (Kilfoy et al., 2009a). THCA has been one of the least deadly human cancers (La Vecchia et al., 2015). Especially papillary THCA, as one subtype of THCA, is often described as "good cancer" due to its relatively beneficial survival rates (Easley et al., 2013). However, there is a rapid increase in the incidence rate of THCA over the past several decades in many countries including China, which has largely caused public concern about THCA (Kilfoy et al., 2009b; Enewold et al., 2009; Du et al., 2014). In recent years, a great progress has been reported to identify genetic changes in THCA, which provides valuable information for making clinical decision (Fagin and Wells, 2016). In our study, the main goal has been to identify novel therapeutic targets and prognosis biomarkers for predicting pathological characteristics and subsequently treating THCA.

Some RNA molecules, such as short microRNAs (miRNAs) and long non-coding RNAs (lncRNAs), are emerging as important regulators of diverse cellular processes, the function of which has been relatively well studied in numerous kinds of tumors (Batista and Chang, 2013; Yates et al., 2013). MiRNAs are a kind of small, endogenous, non-coding RNAs that control gene expression at the posttranscriptional level (Gennarino et al., 2012). They could exert key function of targeting oncogenes and tumor suppressors and regulate important biological processes by coordinately regulating cancer-related pathways (Boscaino et al., 2019). Therefore, miRNAs play crucial roles in cancer initiation, progression and metastasis (Wang et al., 2015). However, a comprehensive analysis of the important miRNAs that control gene expression and the main signaling pathways activated in THCA has been less studied. LncRNAs are a series of non-coding RNAs with a length of >200 nucleotides that play an essential tumor suppressive or oncogenic role (Sui et al., 2016). Many lncRNAs, such as *HOTAIR*, *TUG1*, *XIST,* and *MALAT1*, could promote cancer cell proliferation, invasion and migration, and act as targets for the prognosis and diagnosis of many human diseases, such as bladder cancer (Hu et al., 2017), pancreatic cancer (Qin and Zhao, 2017), breast cancer (Zuo et al., 2017), cervical cancer (Li et al., 2015), and THCA (Huang et al., 2016). More interestingly, lncRNAs often communicate with mRNAs and perform their biological effect *via* competing for shared miRNAs, functioning as competing endogenous RNAs (ceRNAs) (Salmena et al., 2011). With the advent of ceRNA theory, lots of studies have reported that lncRNA, mRNA, and other RNA act as natural miRNA sponges to suppress miRNA function and then affect the occurrence and development of human tumors (Ergun and Oztuzcu, 2015; Qi et al., 2015). For example, lncRNA *NUTF2P3-001* was demonstrated to communicate with *KRAS* by competitively binding to *hsa-mir-3923*. The competing relationships between them lead to the proliferation and invasion of pancreatic cancer (Li et al., 2016). LncRNA *MT1JP* was suggested to regulate the expression of *FBXW7* by competitively binding to *miR-92a-3p*, which play important roles in gastric cancer (Zhang G. et al., 2018). Shao et al. identified dysregulated ceRNA-ceRNA interaction in lung cancer and showed that several ceRNA modules may serve as potential diagnostic biomarkers (Shao et al., 2015). Collectively, these findings show the importance of lncRNA-mediated ceRNA interactions, which may contribute to cancer initiation and progression. In the field of thyroid cancer, numerous studies found that non-coding RNAs play crucial roles in cancer progression. For instance, Guo et al. demonstrated that lncRNA MIAT might inhibit EZH2 expression and promote thyroid cancer cell invasion through the miR-150/EZH2 pathway (Guo et al., 2021). Liu et al. found that lncRNA XIST served as a ceRNA for miR-34a *via* sponging miR-34a, competing with MET for miR-34a binding, and regulating thyroid cancer cell proliferation and tumor growth (Hu et al., 2017).

In this study, we collected THCA samples and investigated the alterations of miRNAs, mRNAs and lncRNAs. Functional enrichment and clustering analysis were conducted for these aberrantly expressed RNAs. Multiple interaction networks among miRNAs, mRNAs and lncRNAs were constructed and hub genes were identified. Besides, we evaluated the prognostic roles of the important miRNAs, mRNAs and lncRNAs in THCA. And the functional modules associated with THCA patients’ prognosis were successfully identified. Importantly, we also estimated the correlations between miRNA-regulated lncRNAs and tumor immune cell infiltration. We hope our molecular biomarkers and functional modules could serve as promising diagnostic biomarkers or therapeutic targets for THCA in the future.

## Materials and methods

### Gene expression profiles

Thyroid cancer (THCA) related gene expression profiles, including mRNAs, lncRNAs and miRNAs were downloaded from Xena browser (https://xena.ucsc.edu/). All expression data were processed with log2 transformation. The genes with missing value in >30% samples were deleted. For calculating Pearson correlation between miRNAs and mRNAs/lncRNAs, we only reserved expression data of 563 samples, including 58 control samples and 505 cancer samples. Clinical information of the 563 samples were also obtained from Xena. Expression data and clinical data were provided in Supplementary Table S1-S3.

### Identification of differentially expressed genes

We calculated the differentially expressed mRNAs, lncRNAs and miRNAs in normal and tumor samples using SAM test. We considered that miRNAs or mRNA with |log2 fold change| >1 or *p*-value <0.01 as differentially expressed, lncRNAs with |log2 fold change| >0.58 or *p*-value <0.01 as differentially expressed. All differentially expressed miRNAs, lncRNAs and mRNAs were used for further analysis.

### Network construction

In this study, two types of networks were constructed, containing negative-correlated network (miRNA-mRNA network, miRNA-lncRNA network) and positive-correlated network (mRNA-lncRNA network). The positive-correlated network consisted by mRNAs and lncRNAs is also named as ceRNA network. Before network construction, we firstly collected pan-cancer miRNA-mRNA interaction from starBase database and calculated miRNA-lncRNA interactions by miRanda algorithm (Li et al., 2014). For the negative network, we mapped all these differentially expressed lncRNAs and mRNAs to the miRNA-mRNA/miRNA-lncRNA interactions and extracted the miRNA-differentially expressed mRNA interactions and miRNA-differentially expressed lncRNA interactions. Then we calculated the Pearson correlation coefficients (PCC) between miRNAs and mRNAs/lncRNAs. If the PCC of a miRNA-mRNA/lncRNA pair was smaller than −0.7, we reserved this miRNA-mRNA pair or miRNA-lncRNA pair. Finally, all pairs with PCC < −0.7 were merged into the miRNA-mRNA or miRNA-lncRNA network.

For the positive network, firstly, we listed all the candidate lncRNA-mRNA pairs by sharing common miRNA targets. Then we calculated the PCCs between lncRNAs and mRNAs. If the PCC of a lncRNA-mRNA pair was greater than 0.7, we reserved this candidate mRNA-lncRNA pair. Finally, all pairs with PCC>0.7 were merged into the lncRNA-mRNA network. Cytoscape software was used to network visualization. The igraph package was used to network analysis.

### Identify closely connected network modules

Molecular Complex Detection (MCODE) plug-in in Cytoscape was performed to find functional modules in miRNA-mRNA network. The MCODE algorithm is based on graph-theoretical analysis, which clusters a given network by topology for finding densely connected regions (Bader and Hogue, 2003).

### Survival analysis

In this study, we collected the clinical information of 563 samples for survival analysis. For single gene analysis, we used the expression data to divide samples into high-expression and low-expression group. For multiple gene analysis, we used risk score model. Briefly, we accumulated the regression coefficient and the expression value of each gene and computed the risk score for each patient as follows:

$$RiskScore=\sum_{i=1}^{n}r_{i}\mathrm{Exp}\left(i\right)$$

Where, n is the number of genes in gene set, $r_{i}$ is the univariate Cox regression coefficient of gene i , and $\mathrm{Exp}\left(i\right)$ is the expression value of gene i for a corresponding patient.

We classified THCA patients into two groups by using the mean risk score as a cut-off. That is, patients with the risk score greater than mean value were classified into high-risk group. Patients with the risk score less than mean value were classified into low-risk group. These high-risk group and low-risk group patients were then used to perform Kaplan-Meier survival analysis. Log-rank test with *p*-value <0.05 was used to generate statistical significance.

### Gene enrichment analysis

Gene enrichment analysis of miRNAs and mRNAs were conducted in this study. For miRNAs, miRwalk (http://zmf.umm.uni-heidelberg.de/apps/zmf/mirwalk2/generetsys-self.html) was used for enrichment analysis. For mRNAs, Pathwax (http://pathwax.sbc.su.se/) was used for enrichment analysis.

### Immune cell infiltration of lncRNAs in LUAD patients

Infiltration estimations for all LUAD patients were downloaded from the TIMER2 database. The potential role of lncRNAs in cell infiltration was estimated by calculating the correlation between lncRNA expressions and infiltration estimation scores. Data was provided in Supplementary Table S4 **.**

## Results

### Identification of differentially expressed miRNAs in THCA

**FIGURE 1:**
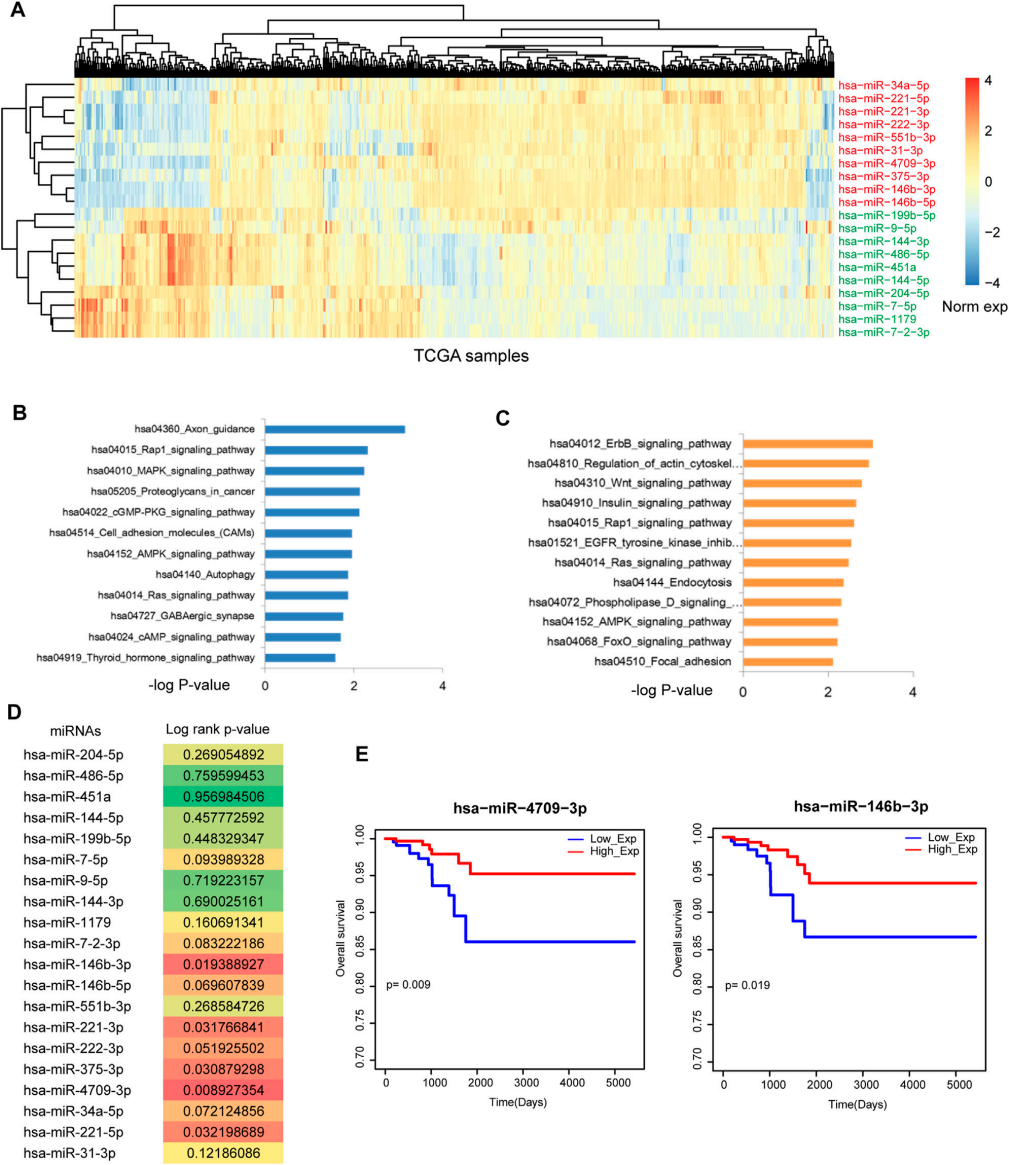
Identification of the potential prognosis biomarker of THCA. **(A)** The heat map of the most upregulated 10 miRNAs and the most downregulated 10 miRNAs. **(B)** Pathway enrichment of the most upregulated 10 miRNAs. **(C)** Pathway enrichment of the most downregulated 10 miRNAs. **(D)** The log-rank *p*-values of the 20 most DE miRNAs. Multiple miRNAs were statistically significant with *p* < 0.05. **(E)** The Kaplan-Meier survival curves of *miR-4709-3p* and *miR-146b-3p*.

Firstly, we downloaded the THCA related miRNA expression profiles from Xena. SAM test was used to identify differentially expressed miRNAs. As a result, 76 differentially expressed miRNAs were identified with strict threshold, including 20 upregulated miRNAs and 56 downregulated miRNAs. Figure 1A showed the top 20 differentially expressed (upregulated and downregulated) miRNAs in THCA. Some known miRNAs were identified, such as *miR-34a-5p* and *miR-9-5p*. In addition, we performed pathway enrichment analysis for upregulated and downregulated miRNAs, respectively (Figures 1B, C). For the upregulated miRNAs, results showed that these miRNAs were implicated in energy metabolism dysfunction, such as Cgmp-PKG signal, AMPK signal Autophagy and cAMP signal. These signals were demonstrated to play key roles in the pathological processes in THCA. For example, cAMP analogs is considered as the effective inhibitor of undifferentiated THCA cell growth (Grassi et al., 2017). Estrogen/ERα induced autophagy is high related with generation of reactive oxygen species, activation of extracellular-signal-regulated kinases (ERK1/2) and the survival/growth of thyroid tumor cells (Fan et al., 2015). Blockade of autophagy could attenuate THCA (Gundara et al., 2015). For the downregulated miRNAs, some crucial pathways were also called out, such as Wnt signal and Focal adhesion. These results showed that dysfunction of miRNAs could lead to the strike of large amount pathways. Thus, we will uncover the regulatory mechanism of miRNA-mediated gene crosstalks.

We detected the prognosis effects of the top differentially expressed miRNAs, results showed that some miRNAs have the strong prognosis effects in THCA (Figure 1D), such as *miR-4709-3p*, *miR-146b-3p* (Figure 1E) and *miR-221-5p*. These results suggested that miRNA-mediated gene regulatory network might have the strong prognosis effects.

### miRNA-mRNA regulatory network

**FIGURE 2:**
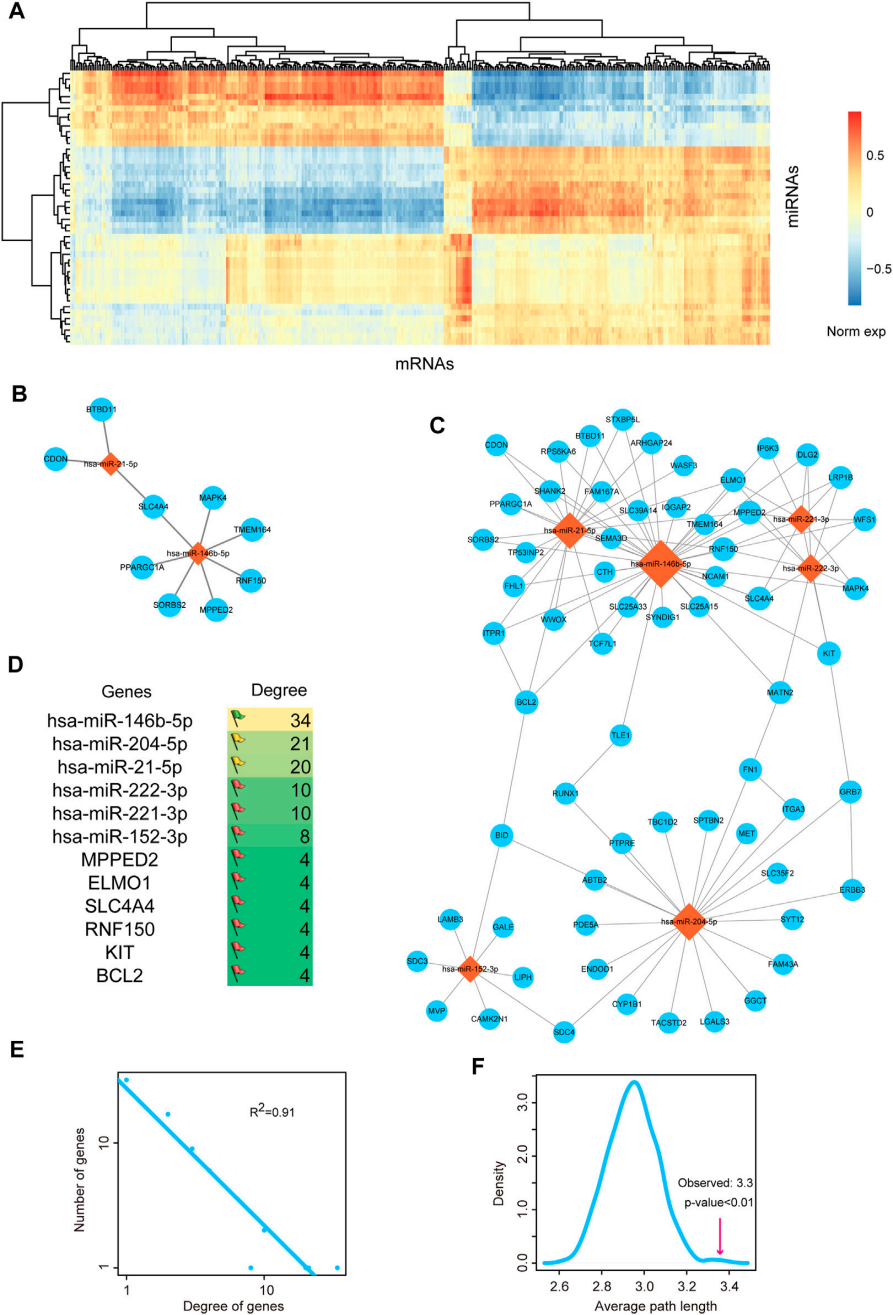
Analysis of miRNA-mRNA network. **(A)** The heat map of correlations between DE miRNAs and DE mRNAs. **(B)** The most negatively expression-correlated 10 DE miRNA-DE mRNA interaction pairs. **(C)** The THCA-related miRNA-mRNA network. Orange diamond represents miRNA and blue circular represents mRNA. Node size represents degree of node. **(D)** The top 6 high-degree miRNA nodes and mRNA nodes of the network. The colors of flags represent the degree of miRNAs. Degree is defined as the edges that link to the nodes. **(E)** Degree distribution of the network. All nodes follow a power-law distribution. This curve indicated that the network was a scale-free network and could be used to identify important nodes. **(F)** Average path length of the real network was significantly shorter than that of 1,000 random networks. The pink line represents the average path length of the real network. Curve was plotted by summarizing the path length distributions of the 1,000 random networks.

Above results showed that miRNA-mediated gene regulatory network might play important roles in the pathological processes of THCA, thus we firstly invested the regulatory network between miRNAs and mRNAs. We calculated the differentially expressed mRNAs based on SAM test. 800 differentially expressed mRNAs were used for further analysis, including 422 upregulated mRNAs and 378 downregulated mRNAs. We detected the global expressive correlation between differentially expressed miRNAs and mRNAs. Results indicated that a sub portion of mRNAs showed negative correlation with miRNAs, implying that these mRNAs were the direct targets of miRNAs (Figure 2A). We extracted the top 10 pairs of high correlated miRNA-mRNA pairs (Figure 2B). The two miRNAs, *miR-21-5p* and *miR-146b-5p*, were reported as the potential therapeutic targets of THCA. *MiR-146b-5p* was demonstrated to regulate TGF signal transduction *via* targeting *SMAD4* in THCA (Geraldo et al., 2012). *MiR-21* and *miR-9* were considered as the potential biomarkers for THCA recurrence (Sondermann et al., 2015). Then, we mapped all these negative miRNA-mRNA pairs into the miRNA-mRNA interaction of starBase database, we only reserved the target pairs into the THCA related miRNA-mRNA network (Figure 2C). This network contained 70 nodes and 110 edges. We listed the top 6 high-degree miRNA nodes and mRNA nodes (Figure 2D). We could see that *miR-146b-5p* had the highest degree, indicating miR-146-5p was the key regulator in THCA pathology. We then calculated the degrees of all the nodes in the network. And the result of network degree distribution showed power law distribution (*R* ^2^ = 0.91, Figure 2E). We also computed average path length of the THCA related miRNA-mRNA network and 1,000 random networks generated by remaining the degree of nodes unchanged. Result showed that the average path length of the real network was significantly larger than that of the random networks (*p* < 0.01, Figure 2F). These results suggested that some local closed modules were located in this network.

### Identification function modules in miRNA-mRNA network

**FIGURE 3:**
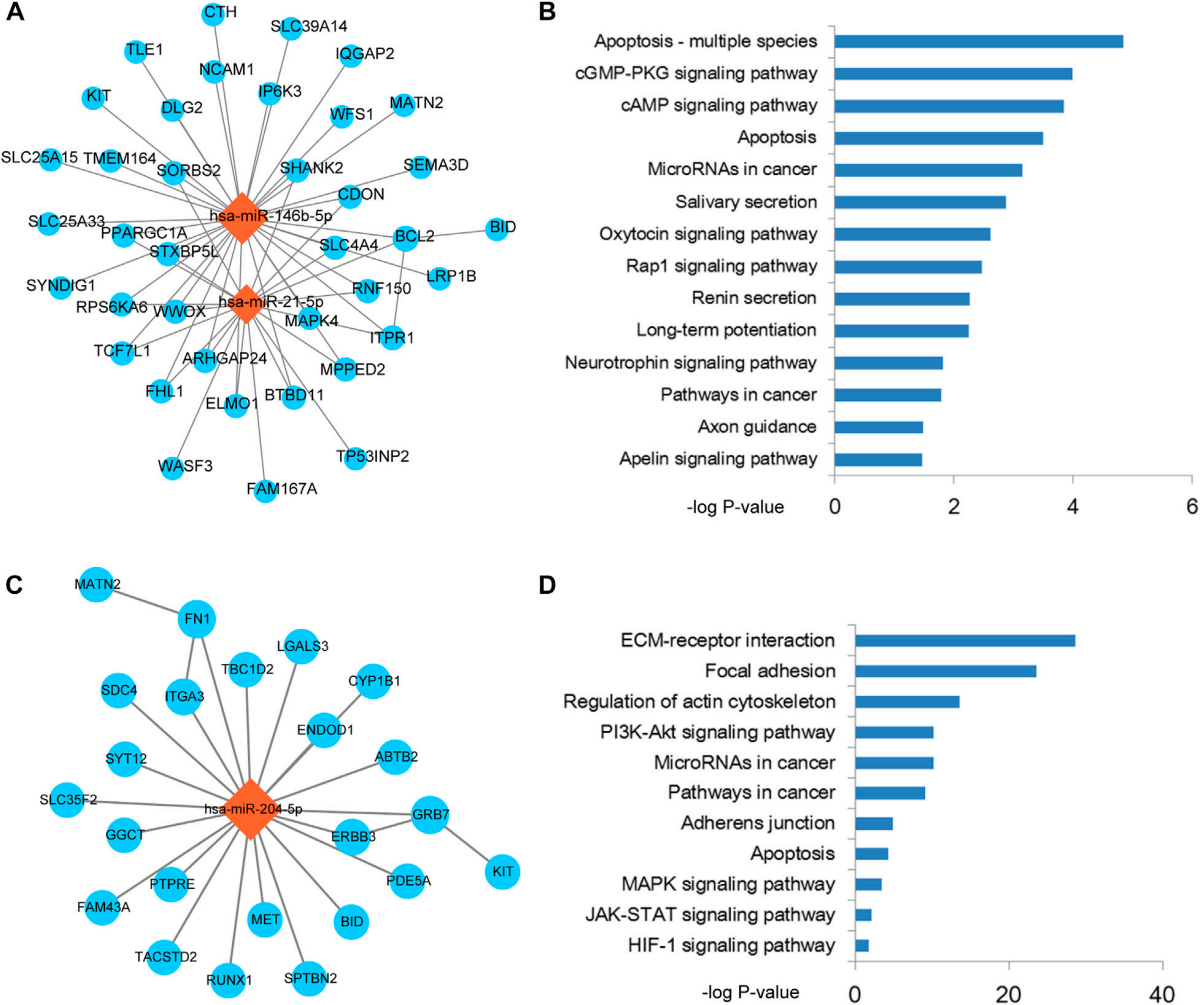
The functional modules of the miRNA-mRNA network. **(A)** Functional module 1 that identified from the miRNA-mRNA network by MCODE. **(B)** Pathway enrichment results of the mRNAs in module 1. **(C)** Functional module 2 that identified from the miRNA-mRNA network by MCODE. **(D)** Pathway enrichment results of the mRNAs in module 2.

Based on the results of previous step, therefore, we performed module analysis for THCA related miRNA-mRNA network. As a result, two modules from the miRNA-mRNA network were identified by MCODE. Pathway enrichment analysis was performed for the two modules. The module 1 was consisted of 2 miRNAs, 38 mRNAs and 56 edges (Figure 3A). Intriguingly, this module was controlled by *miR-21* and *miR-146b-5p*. Pathway results showed that this module was highly related to apoptosis and cGMP/cAMP signal (Figure 3B). As we all known that enhancement of cancer cell apoptosis level could lead to the good prognosis. cGMP and cAMP were also used to cancer drug design. The module 2 was consisted of 1 miRNA, 23 mRNAs and 25 edges (Figure 3C). This module was controlled by *miR-204-5p*, which has been broadly investigated in THCA. Wu et al. demonstrated that *miR-204* was downregulated in THCA and targeted the 3’UTR of *HMGA2*. Overexpression of *miR-204* could inhibit the expression of cyclin D1 and Ki67 and activate the expression of P21, which could subsequently lead to the blockade of THCA cell proliferation (Wu et al., 2019). This module could also regulate the known cancer pathways, such as ECM-receptor interaction, focal adhesion and MAPK signal (Figure 3D). Furthermore, we also performed gene ontology enrichment analysis for genes in module 1 and module 2, respectively (Supplementary Figure S1). Results showed that some key biological processes were enriched in the two modules, such as apoptotic signal, cell migration and adhesion.

### miRNA-lncRNA regulatory network

**FIGURE 4:**
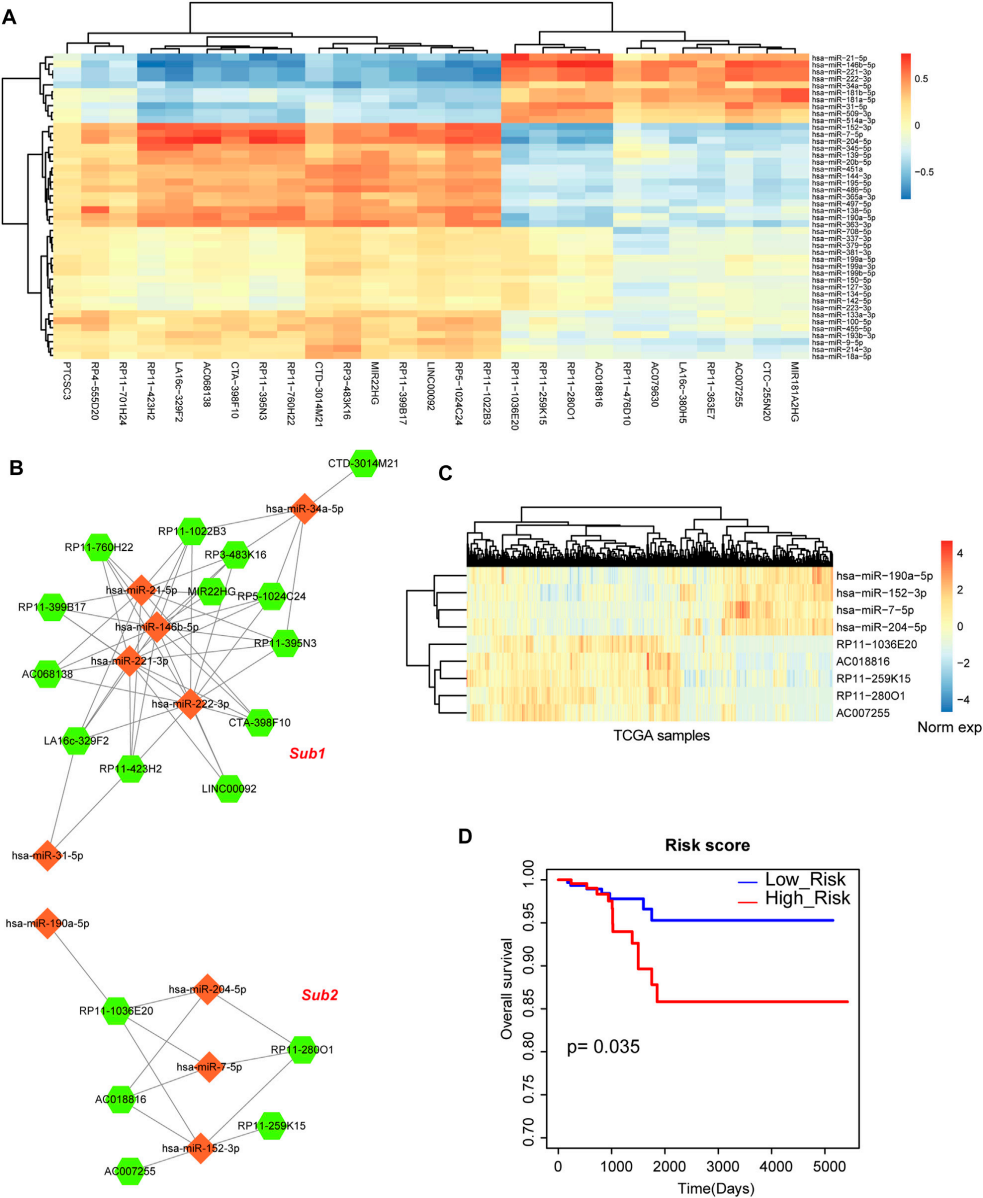
Analysis of miRNA-lncRNA network. **(A)** The heat map of correlations between DE miRNAs and DE lncRNAs. **(B)** The THCA related miRNA-lncRNA network that contains 2 subnetworks. Orange diamond represents miRNA and green hexagon represents lncRNA. Node size represents degree of node. **(C)** The heat map of the expression of subnetwork 2. **(D)** A Kaplan-Meier survival curve of subnetwork 2.

Above results indicated that miRNA-mRNA network played important roles in the biological processes of THCA, thus, we then investigated the regulatory network between miRNAs and lncRNAs. Firstly, we calculated the differentially expressed lncRNAs based on SAM test. 64 differentially expressed lncRNAs were used for further analysis, including 46 upregulated lncRNAs and 18 downregulated lncRNAs. We also detected the global expressive correlation between differentially expressed miRNAs and lncRNAs. Some lncRNAs showed negative correlation with miRNAs, implying that these lncRNAs could sponge these miRNA targets (Figure 4A). Then, we mapped all these negative miRNA-lncRNA pairs into the miRNA-lncRNA interaction of miRanda algorithm, we also only reserved the mapped pairs into the THCA related miRNA-lncRNA network (Figure 4B). This network contained 28 nodes (10 miRNAs and 18 lncRNAs) and 63 edges. In this network, we could see that 2 sub-portions were divided. In subnetwork 1, some miRNAs constituted this network frame, such as *miR-21* and *miR-146b*. However, subnetwork 2 was the new miRNA-mediated network, which was controlled by *miR-204*, *miR-7*, *miR-190* and *miR-152* (Figures 4B, C). Previous studies also demonstrated that some of these miRNAs were the potential targets of THCA (Kang et al., 2017; Liu et al., 2019). These results suggested that miRNA-mediated lncRNAs might participate in the regulation of THCA. Furthermore, survival analysis indicated that subnetwork 2 had the strong prognosis effect (Figure 4D).

### mRNA-lncRNA regulatory network

**FIGURE 5:**
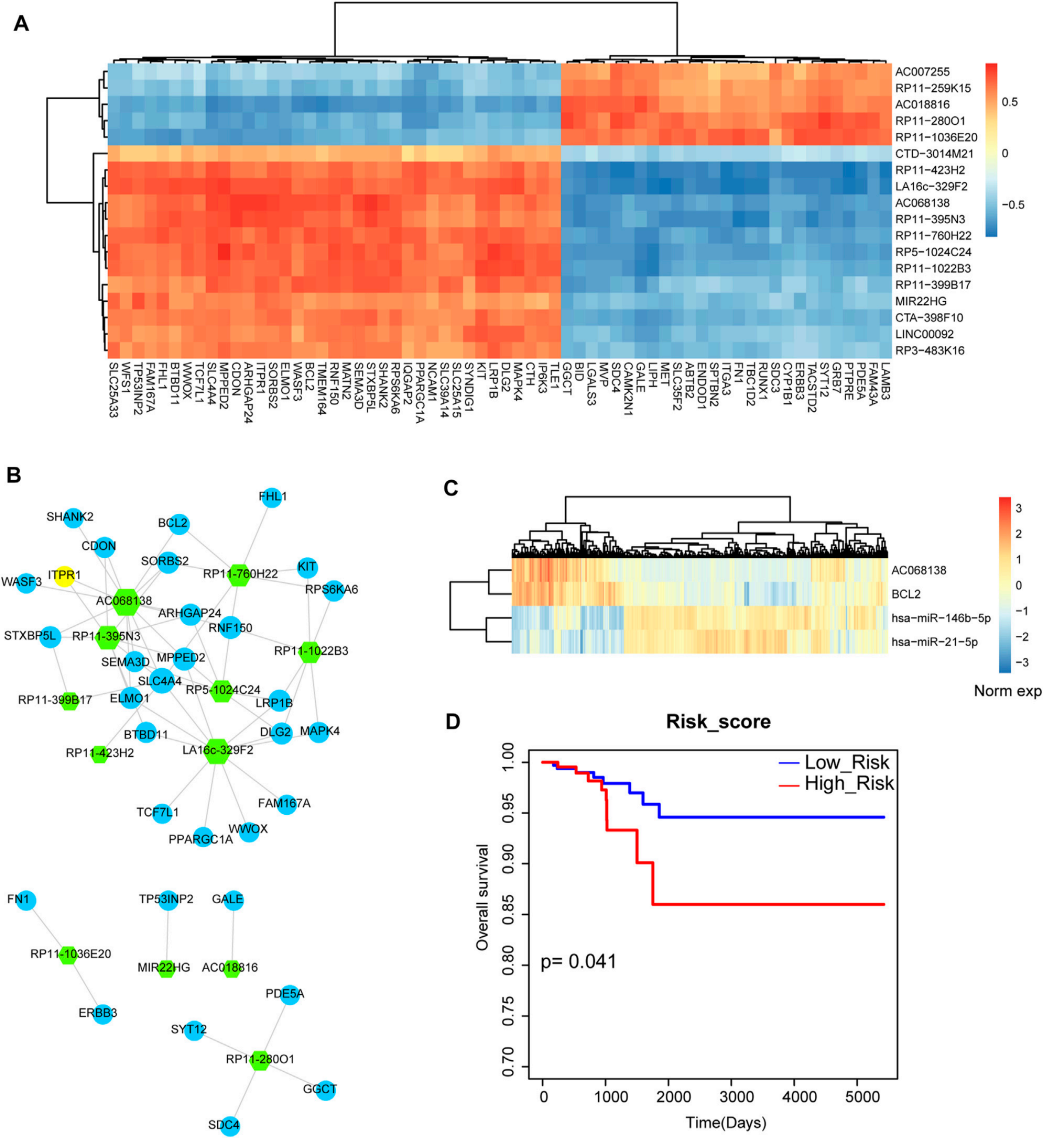
Analysis of lncRNA-mRNA network and lncRNA-miRNA-mRNA crosstalk. **(A)** The heat map of correlations between lncRNAs and mRNAs that shared at least one common miRNA. **(B)** The thyroid cancer-related lncRNA-mRNA ceRNA network. Green hexagon represents lncRNA and blue circular represents mRNA. Node size represents degree of node. **(C)** The heat map of the expression of *AC068138*, *BCL2*, *miR-146* and *miR-21*. **(D)** A Kaplan-Meier survival curve of the combination of *AC068138*, *BCL2*, *miR-146* and *miR-21*.

Next, we want to investigate the miRNA-mediated crosstalk between lncRNAs and mRNAs. We listed all the lncRNA-mRNA pairs based on the strategy of sharing common miRNA targets. We then detected the global expressive correlation between mRNAs and lncRNAs. Results showed that some lncRNAs were high positive correlated with mRNAs, implying that these lncRNAs could form ceRNA pairs with mRNAs (Figure 5A). Then, we mapped all these positive mRNA-lncRNA pairs into the previous miRNA-lncRNA network and miRNA-mRNA network. Based on the strategy of sharing common miRNAs, we merged the mRNA-lncRNA pairs into the THCA related mRNA-lncRNA network (Figure 5B). This network contained 44 nodes and 64 edges. The lncRNA node with the highest degree was *AC068138*. Interestingly, we found that apoptosis gene *BCL2* was the interaction partner of *AC068138*. Then, we listed the miRNA mediators between *AC068138* and *BCL2* (Figure 5C). Results showed that *miR-146* and *miR-21* were the mediator between *AC068138* and *BCL2*, which were demonstrated as the crucial miRNA regulators in THCA. We also detected the prognosis effect for the triple pair, result showed that this triple pair had strong prognosis effect in THCA (Figure 5D). These results suggested that the miRNA-mediated mRNA-lncRNA ceRNA crosstalks might be used as prognosis biomarkers in THCA.

### Immune cell infiltration of lncRNAs in THCA

**FIGURE 6:**
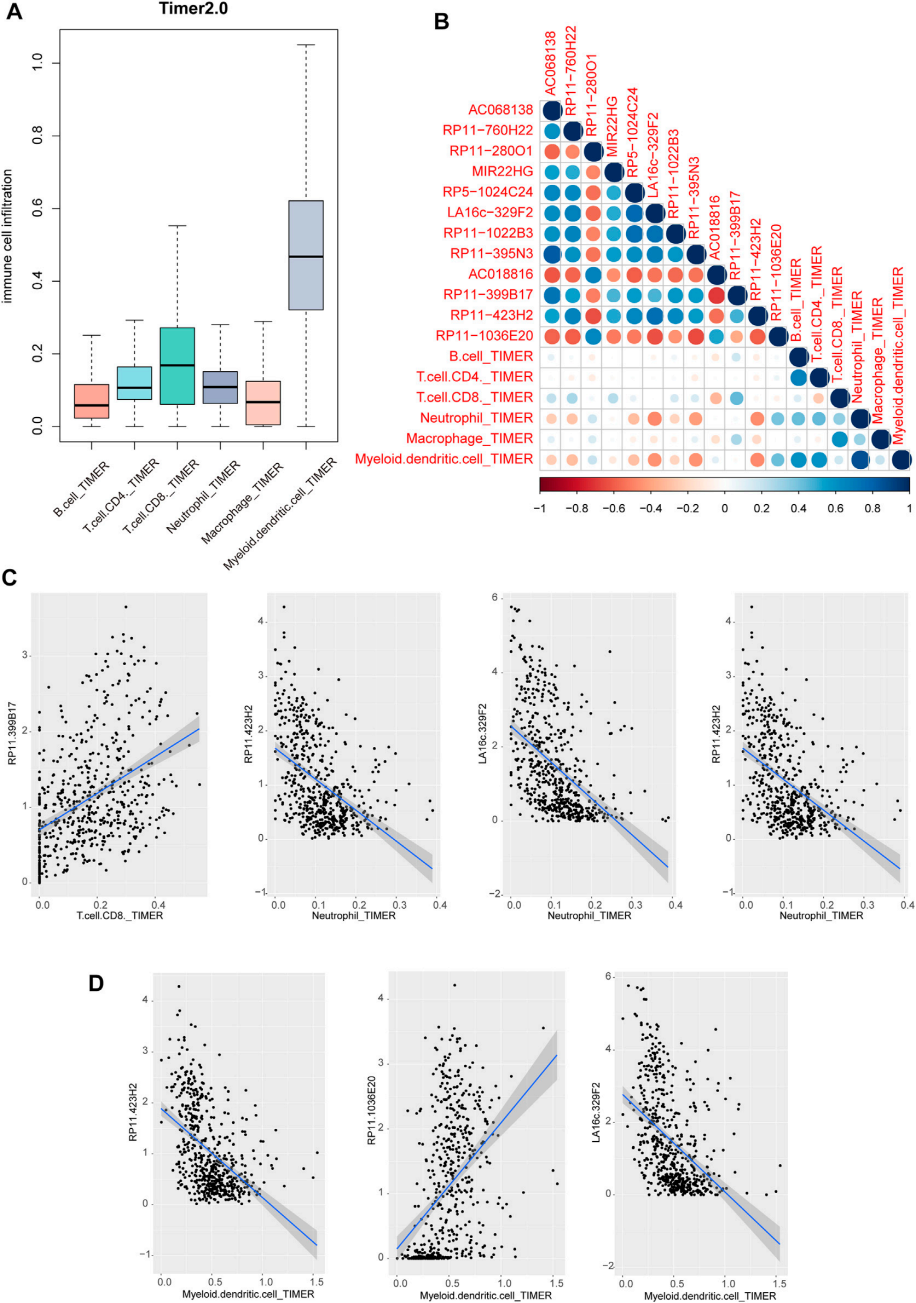
Immune cell infiltration of lncRNAs in THCA. **(A)** Immune cell infiltration levels of six type immune cells of TCGA patients in TIMER 2.0 database **(B)** The correlation heatmap of correlation coefficients between lncRNA expression and immune cell infiltration score. **(C–D)** Scatter plots of correlations between lncRNA expression and TIMER2 immune cell infiltration score.

Previous studies have revealed that immune cell infiltration was existed in cancers, which could be used to develop novel immunotherapy for cancers. We performed lncRNA function enrichment analysis for the differentially expressed lncRNAs *via* LncSEA, results showed that some crucial functions were enriched, including immune, apoptosis and cell proliferation (Supplementary Figure S2) (Chen et al., 2021). Thus, we investigated the regulatory potentials of above lncRNAs in immune cell infiltration. Firstly, we downloaded the pan-cancer cell infiltration data from TIMER2.0 database. Results showed that multiple immune cells were infiltrated in THCA. Obviously, the dendritic cell was the top infiltrated cell type **(** Figure 6A **).** Then, we calculated the correlations between immune cell infiltration levels from TIMER 2.0 and lncRNA expressions. Results showed that neutrophil cell and myeloid dendritic cell were high related to these lncRNAs **(** Figure 6B **)**. From the perspective of lncRNAs, we found that 3 lncRNAs were negatively related to immune cells, including LA16c−329F2, RP11−395N3 and RP11−423H2 **(** Figure 6C **)**. And lncRNA RP11−399B17 and RP11−1036E20 showed a positive correlation with immune cell infiltration **(** Figure 6D **)**. These results suggested that miRNA-regulated lncRNAs might involve in cancer biology by regulating immune cell infiltration levels in THCA.

## Discussion

The incidence of THCA is continuously increasing in the last few years. Although the prognosis of patients with early-stage THCA is favorable, the 5-year survival rate remains troubled (Hundahl et al., 1998). It is urgent to find more effective therapeutic and prognosis targets. According to previous reports, we knew that miRNAs or lncRNAs could regulate progression of multiple cancers including THCA (Zhu et al., 2016; Zhang X. F. et al., 2018; Ding et al., 2018; Yuan et al., 2018). More importantly, some non-coding RNAs were considered as the known prognosis biomarkers in thyroid cancer, such as let-7a, LINC-PINT, H19, MALAT1 and HOXA-AS2 (Chu et al., 2017; Liu et al., 2017; Dai et al., 2019; Jiang et al., 2019; He et al., 2021). Thus, in this study, we identified THCA-specific upregulated and downregulated miRNAs based on TCGA expression profile. Several known THCA-related miRNAs were identified, such as *miR-34a-5p* and *miR-9-5p*. Interestingly, *miR-4709-3p* and *miR-146b-3p* were found to significantly classify high-risk group and low-risk group of THCA patients, suggesting that they may be potential prognosis biomarkers of THCA.

Furthermore, we constructed THCA-related miRNA-mRNA network and miRNA-lncRNA network to better display global properties of these RNA molecules. Network topological feature analysis demonstrated integrative power of miRNAs and their target mRNAs/lncRNAs. Network hub analysis suggested that hub miRNAs or lncRNAs may have the potential to be cancer biomarkers. Functional module analysis showed favorable potential for prediction of THCA prognosis. Survival analysis indicated the closely connected RNAs in the network may function in survival state of THCA patients and have the potential prognosis ability.

Among multiple molecular mechanisms, ceRNA theory is widely acknowledged that lncRNAs can exert biological function in human cancers through acting as miRNA sponges. Thus, in the present study, we constructed a ceRNA network according to the bioinformatics approach. By comprehensively analyzing these ceRNA crosstalk, we revealed many important properties and ceRNA regulation patterns in THCA. Intriguingly, the RNAs in this ceRNA network may be utilized to reveal prognosis of THCA based on our current analytic results.

Distinct immune cell proportions in tumor tissue were confirmed to correlate with the antitumor immunological state in the tumor microenvironment. Tumor immune cell infiltration calculation had also been considered as the key method to unveil the mystery of tumor microenvironment. Previous studies found that some lncRNAs were high related to the immune cell states in thyroid cancer. For instance, Sahin found that lncRNA H19 expression was positively correlated with infiltration level of diverse immune cells, including CD8+T cells, CD4+T cells, neutrophils, B cells, dendritic cells and macrophages. H19 was closely associated with multiple immune markers in THCA (Sahin, 2022). In this study, we evaluated the crosstalks between regulatory lncRNAs and immune cell infiltrations and found that some lncRNAs, such as LA16c-329F2 and RP11-423H2, were high related to multiple immune cell infiltration. Furthermore, we also evaluated the crosstalks between miRNAs/mRNAs and immune cell infiltration, results showed that they were highly correlated (Supplementary Figure S3–4).

In summary, our systematically analysis not only shed new light on the molecular mechanism of tumorigenesis, but also help to tumor prognosis and discovery of therapeutic targets. However, there were some limitations in our study. Firstly, limited data on miRNAs and lncRNAs in THCA are used in this study. If a large number of miRNA and lncRNA expression profiles are available, we may discover more valuable information. Secondly, there are five histological subtypes of THCA, including papillary, follicular, poorly differentiated, anaplastic and medullary. In this study, we only analyzed TCGA expression data containing tumor and normal samples without considering different subtypes of THCA. In the future, when achieving more useful data, we will further focus on significant variability among these different subtypes of THCA. Thirdly, the questions about diagnosis and prognosis of THCA are extremely complicated. The combination of more detailed experimental research could help to deeply understand the THCA-related pathogenesis and molecular mechanism. Finally, we performed MCODE algorithm to identify biological modules, which is depended by network density. Some non-core modules that located in the network border will be ignored.

## Data Availability

The original contributions presented in the study are included in the article/Supplementary Material, further inquiries can be directed to the corresponding author.

## Supplementary material

SUPPLEMENTARY TABLE S1 miRNA expression profile of TCGA THCA patients.

SUPPLEMENTARY TABLE S2 IncRNA expression profile of TCGA THCA patients.

SUPPLEMENTARY TABLE S3 Clinical information data of TCGA THCA patients.

SUPPLEMENTARY TABLE S4 Immunecellinfiltration estimation scores of TCGA THCA patients.

SUPPLEMENTARY FIGURE S1 Gene ontology enrichment of mRNAs in module 1 and module 2.

SUPPLEMENTARY FIGURE S2 Enrichment analysis of THCArelated differentially expressed lncRNAs.

SUPPLEMENTARY FIGURE S3 Immune cell infiltration of mRNAs in THCA.

SUPPLEMENTARY FIGURE S4 Immune cell infiltration of miRNAs in THCA.
