# Supplementary figures and images for: Identification of non-coding RNA related prognosis biomarkers based on ceRNA network in thyroid cancer
Source: Front Genet. 2023 Apr 20;14:1157438. doi: 10.3389/fgene.2023.1157438 (PMC10158935; doi:10.3389/fgene.2023.1157438)

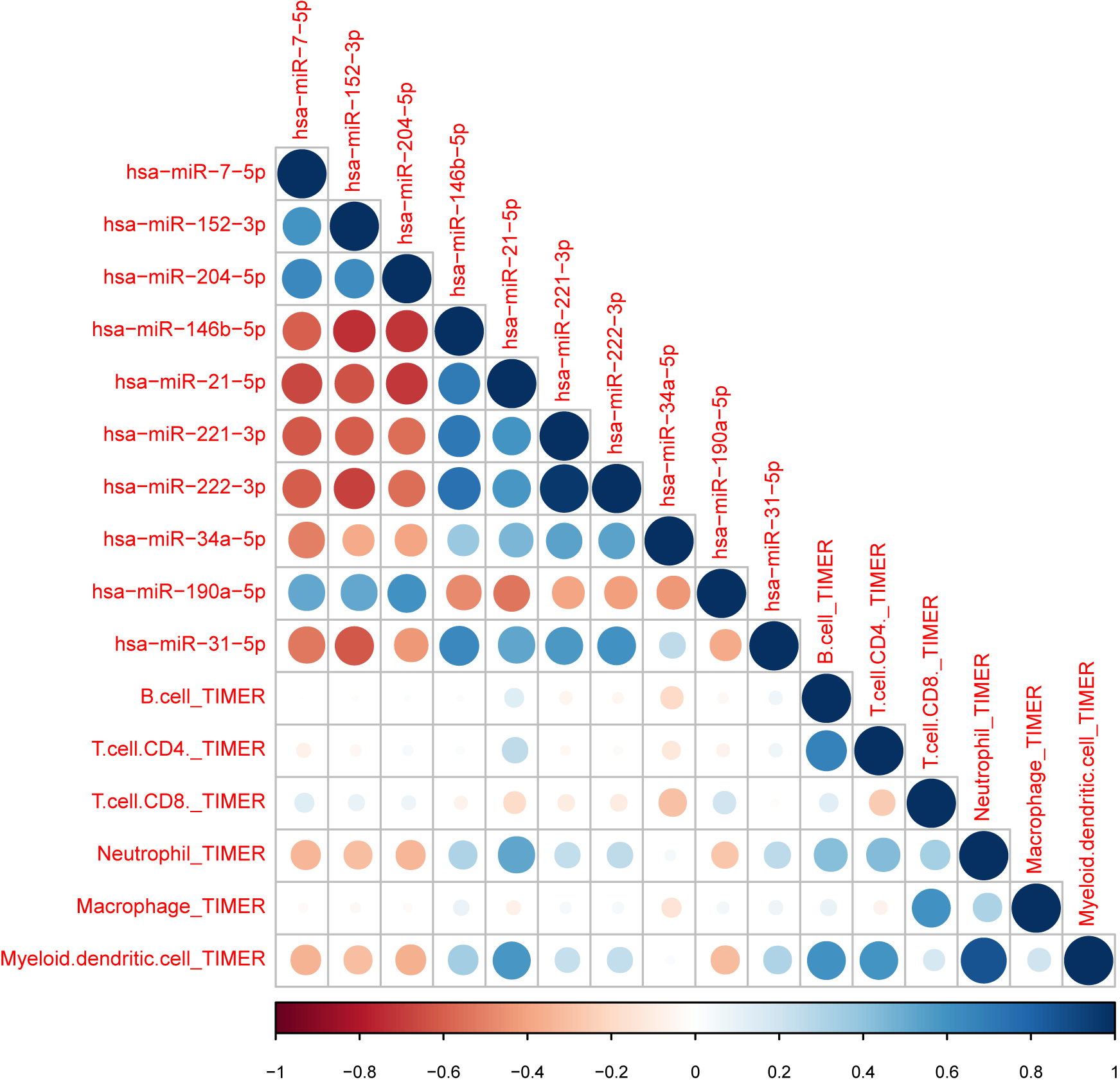

Supplement: Supplementary file 3 [file Image3.TIF]

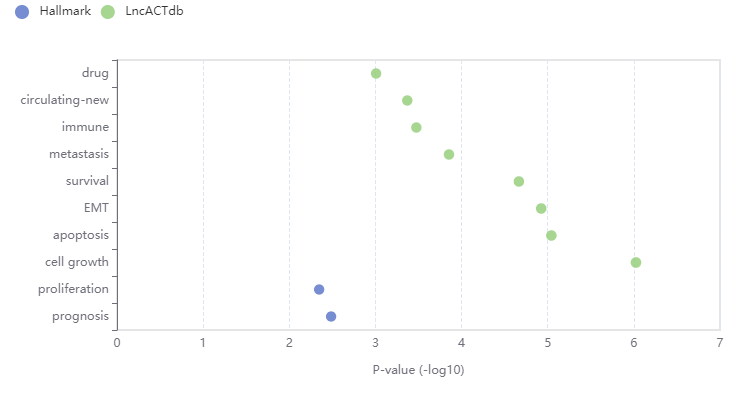

Supplement: Supplementary file 4 [file Image4.TIF]

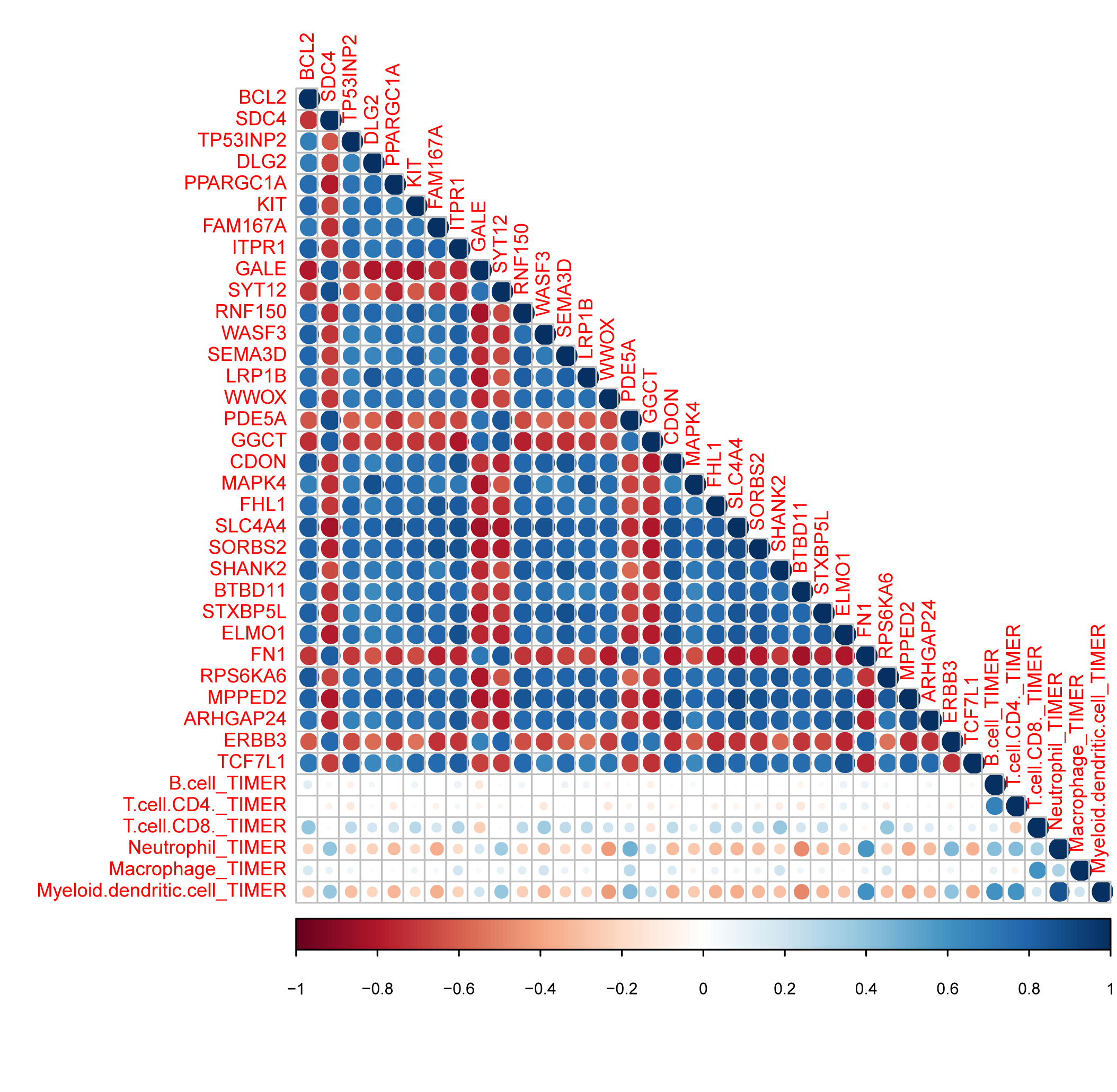

Supplement: Supplementary file 5 [file Image2.TIF]

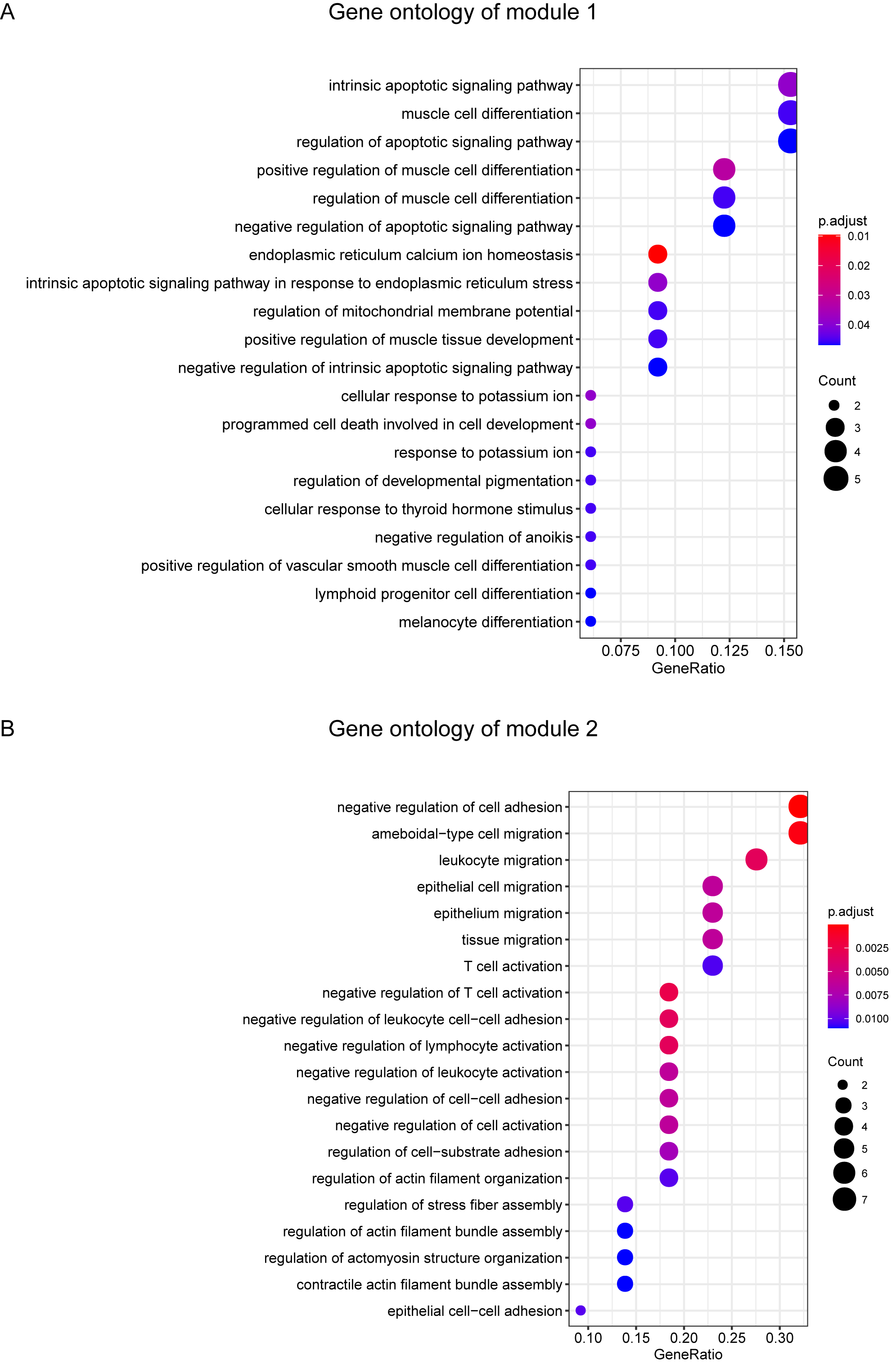

Supplement: Supplementary file 6 [file Image1.TIF]
